# Supplementary material for: Determinants of quality antenatal care use in Kenya: Insights from the 2022 Kenya Demographic and Health Survey
Source: PLOS Glob Public Health. 2024 Sep 19;4(9):e0003460. doi: 10.1371/journal.pgph.0003460 (PMC11412633; doi:10.1371/journal.pgph.0003460)
Supplement: Attachment — Submitted filename: Review Comments][June 22, 2024.doc [file pgph.0003460.s001.doc]

**REVIEWER REPORT**

**TOPIC**

Determinants of quality Antenatal Care utilization in Kenya: insights from the 2022 Kenya Demographic and Health Survey

**Remark(s) 1:** Reads fine.

**ABSTRACT**

**Remark(s) 2:** Reads well.

**INTRODUCTION**

**Page 3, Para 1, Lines 67-69:** “*The absence of quality ANC can have severe consequences, which lead to increased ….*”

**Remark(s) 4:** There is a problem with this sentence, please fix.

**Page 3, Para 2, Lines 74-77:**

**Remark(s) 5:** This paragraph is just two sentences, this is not good enough. I suggest you collapse this and merge with the previous paragraph.

**Remark(s) 6:** On the whole, the section reads well. However, there are issues with the paragraphing throughout the section. There are too many two and three sentence paragraphs which is NOT good enough. The authors must merge most of these paragraphs.

**METHODS**

**Pages 5-7:**

Sampling design, Data collection, and Source

Study variables

Statistical analysis

**Remark(s) 7:** Apart from the “Statistical analysis”, what was the role of the authors in the “Sampling design, Data collection, and Source” and “Study variables”? Were the authors merely reporting how the survey was conducted by KDHS? This section must carefully looked at again to ensure the global audience are not left confused.

**DISCUSSION**

**Page 18, Para 1, Lines 256-259:** “*The difference between our study and the literature may be attributed to the differences in health policies, health facility standards, and ANC implementation challenges in various countries (Idowu et al., 2022)Ajibola Idowu, 2022 & MoH Kenya 2021)*”.

**Remark(s) 8:** There are issues with the citations here (these are flagged in red), please fix.

**Page 20, lines 293 & 294:** “*Therefore, it is recommended to implement interventions….*”

**Remark(s) 8:** There is a problem with the above sentence, please fix.

**CONCLUSION**

Apart from the recommendations for further research, all other recommendations made in the study must be directed at specific organizations or institution (public or private) for action. This should affect similar recommendations made in the discussion.

**REFERENCES**

**Remarks 9:** Some of your references on the main reference list are in APA style and others are not. This must be fixed.

**GENERAL COMMENTS**

Overall, this paper was well written paper and the authors have demonstrated good knowledge of the subject matter. The authors should do well to fix the issues raised to help improve the overall quality of the paper.
