# Supplementary material for: Determinants of quality antenatal care use in Kenya: Insights from the 2022 Kenya Demographic and Health Survey
Source: PLOS Glob Public Health. 2024 Sep 19;4(9):e0003460. doi: 10.1371/journal.pgph.0003460 (PMC11412633; doi:10.1371/journal.pgph.0003460)
Supplement: Attachment — Submitted filename: Response to Reviewers.docx [file pgph.0003460.s002.docx]

| # | Comment | Response | Page/line number |
| --- | --- | --- | --- |
|  | **Reviewer 1** |  |  |
| 1 | Reviewer #1: Overall, this paper was well written paper and the authors have demonstrated good knowledge of the subject matter. The authors should do well to fix the issues raised to help improve the overall quality of the paper. | Thank you very much for the compliment. All issues have been addressed |  |
| 2 | TOPIC  Determinants of quality Antenatal Care utilization in Kenya: insights from the 2022 Kenya Demographic and Health Survey  Remark(s) 1: Reads fine. | Thank you very much for the compliment. |  |
| 3 | ABSTRACT  Remark(s) 2: Reads well. | Thank you very much for the compliment. |  |
|  | **INTRODUCTION** |  |  |
| 4 | Page 3, Para 1, Lines 67-69: “The absence of quality ANC can have severe consequences, which lead to increased ….”  Remark(s) 4: There is a problem with this sentence, please fix. | Thank very much for your feedback, the sentence has been grammatically revised. The sentence has been revised to “*The provision of low-quality ANC leads to an increased risk of maternal and newborn mortality and morbidity*.”  Above all the whole paper has been grammatically revised by a professional English editor (Ms. Audrey Holmes) |  |
| 5 | Page 3, Para 2, Lines 74-77:  Remark(s) 5: This paragraph is just two sentences, this is not good enough. I suggest you collapse this and merge with the previous paragraph.  Remark(s) 6: On the whole, the section reads well. However, there are issues with the paragraphing throughout the section. There are too many two and three sentence paragraphs which is NOT good enough. The authors must merge most of these paragraphs. | Thank very much for your feedback*. Paragraphs 1 and 2 have been combined, as well as paragraphs 3 and 4.* | Pg 3, LN 66-93 |
| 6 | **METHODS**  Pages 5-7:  Sampling design, Data collection, and Source  Study variables  Statistical analysis  Remark(s) 7: Apart from the “Statistical analysis”, what was the role of the authors in the “Sampling design, Data collection, and Source” and “Study variables”? Were the authors merely reporting how the survey was conducted by KDHS? This section must carefully looked at again to ensure the global audience are not left confused. | Thank very much for your feedback. It is important to note that since this manuscript reports findings of secondary data analysis, the authors had no role in Sampling design and Data collection. However, the authors requested the datasets for analysis from **Measure DHS** , selected appropriate variables for analysis, cleaned the dataset, and analyzed the data. This role has been emphasized in the paper, under “Sampling design, Data collection, and Source.” | P9 5, LN 108-112 |
| 7 | **DISCUSSION**  **Page 18, Para 1, Lines 256-259:** “*The difference between our study and the literature may be attributed to the differences in health policies, health facility standards, and ANC implementation challenges in various countries (Idowu et al., 2022)Ajibola Idowu, 2022 & MoH Kenya 2021)*”.  **Remark(s) 8:** There are issues with the citations here (these are flagged in red), please fix.  **Page 20, lines 293 & 294:** “*Therefore, it is recommended to implement interventions….*”  **Remark(s) 8:** There is a problem with the above sentence, please fix. | Thank very much for your feedback. The repetitions in the citation have been removed.  All the citations/references have been addressed and cited in accordance to the journal requirements. The sentence has been grammatically revised. Above all the whole paper has been grammatically revised by a professional English editor-(Ms. Audrey Holmes)  The sentence has revised to:  “Therefore, interventions by various health and non-health stakeholders that promote shared decision-making and autonomy in healthcare-seeking behaviors among pregnant women and their partners are recommended*”* | PG 18, LN 270-272, PG 19, LN 296-298 |
| 8 | **CONCLUSION**  Apart from the recommendations for further research, all other recommendations made in the study must be directed at specific organizations or institution (public or private) for action. This should affect similar recommendations made in the discussion. | Thank you very much for your advice. Although the stakeholders that the study recommends were implied, all recommendations made in the discussion and conclusions have directed to relevant entities. |  |
| 9 | **REFERENCES**  **Remarks 9:** Some of your references on the main reference list are in APA style and others are not. This must be fixed. | Thank very much for your feedback. All the citations/references have been addressed and cited in accordance to the journal requirements. |  |
|  | **Reviewer 2** |  |  |
| 1 | Reviewer #2: The findings highlight persistent gaps in ANC quality despite efforts by the Ministry of Health, underscoring the need for targeted interventions to enhance healthcare provider training, community awareness, and adherence to ANC guidelines to improve maternal and newborn health outcomes. | Thank very much for your compliment. |  |
| 2 | The authors need to edit the grammar | Thank you very much for the constructive feedback. The whole paper has been grammatically revised by a professional English editor-(Ms. Audrey Holmes) |  |
|  | ."Ethiopia having a prevalence of 31.3%, Uganda at 61.4%, and Nigeria at 45%" please put this in order of percentages | Thank you very much for the constructive feedback. This has been put in order and it now reads “*having a prevalence of 31.3%, Nigeria at 45%, and Uganda at 61.4%”* | Pg 3, LN 70-71 |
| 3 | The authors need to revise the keywords and make them meaningful | Thank you very much for the constructive feedback. These have been revised in line with our study and standard MESH words | PG 3, LN 60 |
| 4 | line 108, explain what ICF is | Thank you very much for the constructive feedback. The term has been written in full as Inner-City Fund (ICF) on first use and used as an abbreviation in the later part of the manuscript. | PG 4, LN 103 |
| 5 | Explain about the study setting | Thank very much for your feedback. Since demographic and Health Surveys (DHS) are nationally-representative household surveys data was obtained from all regions of Kenya, thus it may be difficult to specify a specific study setting. However, the term nationally representative sample has been added to the methodology section. | PG 4, LN 102 |
| 6 | The authors need to make their discussion more stronger, it doesnt fully discuss the results | Thank you very much for the constructive feedback. We tried to discuss all findings of our multivariate analysis as a best practice. Discussing results of bivariate analysis may be confusing to the readers, especially in situations where multivariate analysis has been conducted. Nevertheless, we have made improvements to discussion through grammatically editing manuscript, emphasizing key points and directing all our recommendations to particular authorities and organizations. | PG 16, LN 226-279 |
